# Hypovitaminosis D in recent onset rheumatoid arthritis is predictive of reduced response to treatment and increased disease activity: a 12 month follow-up study

**DOI:** 10.1186/s12891-015-0505-6

**Published:** 2015-03-15

**Authors:** Manuela Di Franco, Ilaria Barchetta, Cristina Iannuccelli, Maria Chiara Gerardi, Silvia Frisenda, Fulvia Ceccarelli, Guido Valesini, Maria Gisella Cavallo

**Affiliations:** Rheumatology Unit-Department of Internal Medicine and Medical Specialties, Sapienza University of Rome, Viale del Policlinico 155, Rome, 00161 Italy; Internal Medicine Unit-Department of Internal Medicine and Medical Specialties, Sapienza University of Rome, Viale del Policlinico 155, Rome, 00161 Italy

**Keywords:** Rheumatoid arthritis, Vitamin D, Disease activity, Musculoskeletal ultrasonography, Response to treatment

## Abstract

**Background:**

Vitamin D displays immunomodulatory activities and has been proposed as a potential player in the pathogenesis of rheumatoid arthritis (RA). A negative association between serum 25(OH) vitamin D levels and RA activity was demonstrated but longitudinal studies investigating the role of vitamin D levels in predicting RA activity and response to treatment are lacking. Therefore, this study was designed to test the hypothesis of an association between serum 25(OH) vitamin D levels at RA diagnosis and disease activity evaluated by clinimetric, laboratory and ultrasound (US) parameters and to detect the prevalence of remission and response to treatment after 12 months follow-up.

**Methods:**

This is a longitudinal, retrospective study on data obtained from thirty-seven patients with early RA treatment-naïve. Serum inflammatory markers, auto-antibodies and 25(OH) vitamin D levels were obtained at baseline. Hypovitaminosis D was diagnosed for 25(OH) vitamin D levels < 20 ng/ml. Tender joint count (TJCs), swollen joint count (SJCs), Visual Analog Scales (VAS), Disease Activity Score (DAS) 28 score were assessed at baseline and 12 months after diagnosis. Joints synovitis and power-Doppler were evaluated at baseline and 12 months later.

**Results:**

At baseline mean 25(OH) vitamin D levels were 24.4 ± 11.9 ng/ml; 35% of study subjects had hypovitaminosis D which strongly associated with higher RA activity and lower prevalence of remission and response to treatment (all p-values < 0.001). The percentage of patients not presenting a reduction of the US synovitis score after 12 months from diagnosis was significantly higher among patients with hypovitaminosis D than in those with normal serum 25(OH) vitamin D at baseline.

**Conclusions:**

In patients with early RA and basal hypovitaminosis D after 12 months follow-up reduction of disease activity and percentage of remission and response to treatment were significantly lower than those observed in patients with normal vitamin D levels. These results provide further support to the immunomodulatory action of vitamin D in RA and suggest a role of basal vitamin D status in the prediction of disease evolution. Vitamin D measurement and possibly vitamin D supplementation should be considered an additional option in the management of early RA patients.

**Electronic supplementary material:**

The online version of this article (doi:10.1186/s12891-015-0505-6) contains supplementary material, which is available to authorized users.

## Background

Rheumatoid arthritis (RA) is a chronic autoimmune disease characterized by joint involvement and systemic features, leading to a progressive disability and early death [1]. It affects approximately 1% of the population, with higher prevalence in women. Although its aetiology is still unknown, it has been theorized that in genetically susceptible individuals environmental factors trigger an autoimmune response resulting in synovial hypertrophy and chronic joint inflammation and destruction associated with potential extra-articular manifestations [2]. It has been demonstrated that the active form of vitamin D, the 1,25-dihydroxyvitamin D (1,25(OH)_2_D), besides its role in calcium and bone homeostasis, acts as a modulator of immune cells such as macrophages, dendritic cells and activated T cells, expressing its specific vitamin D receptor (VDR) [3,4]. Furthermore, 1,25(OH)_2_D displays an anti-inflammatory role also acting on Th17 cells, contributing to the maintenance of the immunological homeostasis [5-7]. In murine models 1,25(OH)_2_D was able to prevent the onset and progression of arthritis and VDR deficient mice (VDR−/−) showed a more aggressive disease [8,9]. Some epidemiological studies showed a higher prevalence of vitamin D deficiency among patients affected by RA compared with healthy population [10-15], whereas other investigations did not confirm these findings [16,17]. Furthermore, a recent meta-analysis demonstrated that low vitamin D intake is associated with high risk of RA and that a negative association exists between serum vitamin D levels and RA activity [18]. Likewise, evidence about the relationship between hypovitaminosis D and early RA are contrasting [19-21].

Interestingly, an intervention trial conducted in a population of patients with early RA and disease-modifying anti-rheumatic drugs (DMARDs)-naïve showed that trimestral calcitriol supplementation along with triple DMARDs therapy resulted in significant higher pain relief compared with treatment with triple DMARDs plus calcium [14].

At the best of our knowledge, longitudinal studies investigating the role of vitamin D levels at RA diagnosis on the prediction of disease’s activity, remission and response to treatment are missing. Therefore, aims of this study were to test the hypothesis of an association between serum 25(OH) vitamin D levels at the diagnosis of RA and: 1- the disease’s activity evaluated by clinimetric, laboratory and ultrasound (US) parameters, 2- the prevalence of remission and response to treatment in a population of patients affected by early RA naïve for DMARDs treatment, after 12 months of follow-up.

## Methods

### Study population

This is a longitudinal observational retrospective study with a 12 months follow-up. Data from seventy-six consecutive patients referring to the Early Arthritis Clinic, Rheumatology Unit, Sapienza University of Rome, for recently onset RA between 2010 and 2012 were initially obtained. Among them, we enrolled subjects which fulfilled the following inclusion criteria: age between 30 and 65 years, subjects meeting the American College of Rheumatology/European League Against Rheumatism (ACR/EULAR) criteria for RA diagnosis [22], mean disease duration ≤ 24 weeks, BMI 16–25 kg/m^2^, conscious acceptance of written informed consent. The exclusion criteria were: treatment with vitamin D and/or calcium supplementation or drugs affecting the bone and mineral metabolism, multivitamin supplementation, oral contraceptives, parathyroid hormone, L-tyroxine, diuretics, DMARDs, anti-epilepsies and steroids; intestinal malabsorption; diseases associated with hypercalcaemia (lymphoma, sarcoidosis, tuberculosis infection, primary hyperparathyroidism); acute/chronic kidney failure; acute/chronic hepatic failure; type 1 and type 2 diabetes mellitus; metabolic syndrome; history of drugs and/or alcohol abuse. Among the initial seventy-six patients, thirty-seven subjects were considered eligible for the study: fifteen patients were excluded for treatment with drugs affecting the bone and mineral metabolism, eight for multivitamin supplementation, one for oral contraceptives, three for treatment with steroids, one for intestinal malabsorption, two for chronic kidney failure, five for diabetes mellitus, three for metabolic syndrome and one refused to participate.

Study population underwent 12 months follow-up (mean ± SD: 54 ± 3 weeks) during which they were treated with low doses of corticosteroids (Prednisone < 10 mg/day) and methotrexate (7.5-15 mg/week) and attended to quarterly recall visits for clinical evaluation.

### Laboratory tests

For each patient, we collected and analyzed data at baseline (disease onset) and after 12-months follow-up on medical history, physical examination and blood immune-inflammatory parameters, such as: erythrocyte sedimentation rate (ESR) (normal values: ≤15 mm/h in men and ≤20 mm/h in female), C-reactive protein (CRP) level (normal values: <3 mg/L), using standard laboratory methods and to detect IgM-Rhematoid Factor (RF) by nephelometric method (N latex FR, Bering, CV < 4%) and anti-citrullinated protein antibodies (ACPA) by immune-enzymatic method (ImmunoCap, Phadia, <5%). A titre of IgM-RF >15 IU/ml was considered positive, the cut-off point for ACPA positivity was > 12 IU/ml, according to the manufacturer’s instructions.

In order to evaluate vitamin D status in our population at the diagnosis of RA, serum levels of 25(OH) vitamin D (ng/ml), the most stable circulating form of this molecule were also measured [23,24]. In order to minimize the impact of seasonality on circulating vitamin D concentrations, all participants underwent fasting blood sampling for serum 25(OH) vitamin D measurement during autumn-winter period. Vitamin D was measured by immunochemiluminescent method (LIAISON, DiaSorin, CV < 5%) on sera frozen immediately after separation and stored at −25°C for few days.

### Clinical assessment

Clinimetric assessment, routinely used in clinical practice and Clinical Trials to evaluate disease activity and response to treatment, was performed by a rheumatologist experienced in clinimetric evaluation and blinded to laboratory data. At baseline and 12 months of follow up we collected data on: the number of tender and swollen joints on the basis of the 28-joint count and 44-joint count, Ritchie index (a graded assessment of 26 joint regions), visual analogue scale (VAS 0–100 mm) for pain and patient’s disease activity, examinator’s global disease activity assessment (EGA), patient global health assessment (GH) and 28-joints Disease Activity Score (DAS28) [25].

At 12 months follow-up, EULAR response was calculated from the DAS28 scores. Study population was divided in three EULAR response groups: no response, moderate response or good response. To be a good responder, a patient had to show an improvement of at least 1.2 units and achieve an absolute score of < 3.2. Non-responders had to show an improvement of < 0.6, or > 0.6 and ≤ 1.2, and have a final DAS28 score of > 5.1. Moderate responses fell in-between these criteria [26].

### Musculoskeletal ultrasonography

US evaluation was performed both at baseline and after a 12-months follow-up. A systematic multiplanar grey-scale and powerDoppler (pD) examination of 17 joints [wrist, metacarpophalangeal (MCP) joints, proximal interphalangeal (PIP) joints, knee and metatarsophalangeal (MTP) joints bilaterally] was performed according to standard EULAR guidelines, by MyLab70 XVision Gold (Esaote, Italy) machine equipped with a multifrequency linear probe (6–18 MHz); powerDoppler PFR (peak frequency ratio) was 750 Hz, Doppler frequency was 7.5 MHz. Synovial effusion (SE), synovial hypertrophy (SH) and bone erosions were assessed according to the Outcome Measures in Rheumatoid Arthritis Clinical Trial (OMERACT) definitions [27]. US-detected elementary lesions (SE, SH and pD signal) were scored according to a semiquantitative scale based on the severity of the US modifications (0 = normal; 1 = mild; 2 = moderate; 3 = severe). The total synovitis score was obtained from the sum of the all US modifications identified in all the joints. Bone erosions were assessed with a semiquantitative score as follows: grade 0 = no erosion, grade 1 = 1 erosion, grade 2 = 2 erosions, grade 3 ≥ 3 erosions.

### Statistics

SPSS version 17 statistical package was used to perform the analyses. Non-parametric Mann–Whitney test for mean comparison between two independent groups and Wilcoxon test for mean comparison between two related groups were used. The χ-squared test was used to compare the rate of responders to treatment at 12 months follow-up (moderate + good response vs non response) among patients with and without basal hypovitaminosis D. Bivariate correlation analyses were performed by Spearman’s coefficient calculation. Study population was divided in two subgroups according to vitamin D status considering serum 25(OH) vitamin D < 20 ng/ml as a cut-off for insufficient vitamin D levels [28,29]. At the best of our knowledge, clinical remission and response to treatment by DAS28 after a 12 months follow-up in relation to vitamin D status at RA onset have never been studied before. Therefore, in order to confirm the statistical power of this study, we performed a post-hoc sample size calculation considering a prevalence of remission in the subgroup with normal vitamin D status of 68% and of 16% in patients with hypovitaminosis D; thus, we obtained that eleven patients per subgroup were enough to reach the statistical significance with a power of 80% (α error = 0.05, β error = 0.20). For all the above, a p-value < 0.05 was considered statistically significant. Study protocol was reviewed and approved by the Ethics Committee of Policlinico Umberto I, Sapienza University of Rome and conducted in conformance with the Helsinki Declaration. Written consent was obtained from all patients before the study.

## Results

The study included 37 early RA patients. These were mostly female (6 M/31 F) with a mean (SD) age of 47 ± 12 years and median disease duration (SD) of 24 ± 13 weeks. Clinical, biochemical and US characteristics of study population at baseline and after 12 months from diagnosis are shown in Table 1.

**Table 1:** **Clinimetric, laboratory and ultrasound characteristics of study population at baseline and after 12 months follow-up**

**Patients (N = 37)**	**Baseline**	**12 months follow-up**	**P-value**
ESR (mm/h), mean ± SD	31 ± 21	13.9 ± 11	0.0001^
CRP (g/L), mean ± SD	13.6 ± 16	5.81 ± 4.71	0.003^
RF positivity, number (%)	31 (83)	31 (83)	-
APCA positivity, number (%)	30 (81)	30 (8)	-
Morning stiffness (minutes), mean ± SD	62 ± 48	20.8 ± 23	0.0001^
Ritchie index, mean ± SD	10 ± 8.2	2.5 ± 2.9	0.00001^
VAS pain (mm), mean ± SD	63 ± 24	31.9 ± 32	0.002^
VAS disease patient (mm), mean ± SD	58 ± 23	31 ± 27	0.0001^
EGA (mm), mean ± SD	54 ± 20	24.3 ± 20	0.0001^
Number of tender joints (0–44), median (range)	12 (0–35)	2 (0–19)	00001^
Number of swollen joints (0–44), median (range)	5 (0–22)	3 (0–21)	0.0001^
Number of tender joints (0–28), median (range)	8 (0–24)	4 (0–30)	0.0001^
Number of swollen joints (0–28), median (range)	5 (0-20	5 (0–32)	0.0001^
DAS 28 score, mean ± SD	5.18 ± 1	2.77 ± 1.14	0.0001^
Ultrasound Total Synovitis score, mean ± SD	25.7 ± 21.7	8.31 ± 6.2	0.0008^
Ultrasound Total power Doppler score, mean ± SD	3.3 ± 5.9	0.65 ± 1.22	0.005^
Ultrasound Total Erosion score, mean ± SD	1.34 ± 2.7	10.31 ± 9.8	ns^
Patients ≥1 erosion, number (%)	12 (32%)	18 (48%)	ns*
X-Ray Erosion, +ve, number (%)/%	6 (16%)	-	

Results are shown as mean ± SD, median(min-max) or number(percentage) of patients, as appropriated. ^Wilcoxon test applied, *chi-squared test applied. P-value <0.05 are considered significant. ESR, erythrocyte sedimentation rate; CRP, C-reactive protein; RF, Rheumatoid Factor; ACPA, anti-citrullinated protein antibodies; VAS, visual analogue scale; EGA, examinator’s global disease activity assessment; DAS28, 28-joint disease activity score.

### Baseline characteristics

Study population had mean serum 25(OH) vitamin D concentrations of 24.4 ± 11.9 ng/ml; notably, 35% of our sample was affected by hypovitaminosis D at the time of RA diagnosis. Serum 25(OH) vitamin levels were not associated with basal DAS28. In order to characterize our patients according to vitamin D status, we stratified the study sample in two subgroups in relation to the vitamin D balance: patients affected by hypovitaminosis D (25(OH) vitamin D ≤ 20 ng/ml) and with sufficient vitamin D levels (25(OH) vitamin D > 20 ng/ml).

Baseline clinimetric, biochemical and US characteristics did not differ significantly between the two subpopulations although there was a trend in increased prevalence of ACPA and RF positivity and a higher US-pD score in patients affected by hypovitaminosis D compared with subjects with normal 25(OH) vitamin D levels. Furthermore, US synovitis joint score was significantly associated with DAS28 (Spearman’s coefficient = 0.5, p = 0.003).

### 12 months follow-up characteristics

After 12 months from RA diagnosis in the overall population there was a significant reduction of disease’s activity, as expressed by ESR, serum CRP levels, TJCs, SJCs and DAS28, and US synovitis and p-D score (all p-values < 0.001), as expected (Table 1).

Among the whole study population, individuals with hypovitaminosis D at baseline had significantly higher disease activity at the 12 months follow-up evaluated by clinimetric index, compared with patients with sufficient 25(OH) vitamin D levels. Furthermore, ESR, CRP and US inflammatory and US bone erosion scores were increased in presence of hypovitaminosis D although these differences did not reach the statistical significance. Clinimetric, laboratory and US characteristics of study population according to vitamin D status at baseline and 12 months follow-up are shown in Table 2.

**Table 2:** **Characteristics of study population at baseline and after 12 months follow-up according to vitamin D status at the time of RA diagnosis**

**Parameters**	**Baseline**			**12 months follow up**		
	***25(OH)D <20 ng/ml (N = 13)***	***25(OH)D >20 ng/ml (N = 24)***	***P-value***	***25(OH)D <20 ng/ml (N = 13)***	***25(OH)D >20 ng/ml (N = 24)***	***P-value***
Age, mean *±* SD	51.3 ± 13.1	46.0 ± 11.2	ns^	-	-	-
Sex, M/F	2/11	3/22	ns*	-	-	-
BMI (Kg/m^2^), mean *±* SD	25.5 ± 5.2	25.5 ± 4.2	ns^	-	-	-
Vitamin D (ng/ml), mean *±* SD	12.4 ± 3.4	30.4 ± 9.9	0.0001^	-	-	-
Disease Duration (weeks), mean *±* SD	21.6 ± 12.8	23.8 ± 18.1	ns^	-	-	-
Morning stiffness (minutes), mean ± SD	56.6 ± 49.4	83.6 ± 102.2	ns^	38.3 ± 27.2	12.1 ± 15.8	0.002^
ESR (mm/h), mean ± SD	25.9 ± 13.1	35.5 ± 25.4	ns^	15 ± 10	13.4 ± 12.9	ns^
CRP (g/L), mean ± SD	8.2 ± 8.2	16.4 ± 19.3	ns^	5.1 ± 4.2	4.1 ± 4.7	ns^
RF positivity, number (%)	11 (85%)	20 (83%)	ns *	9 (69%)	13 (54%)	0.002*
APCA positivity, number (%)	11 (85%)	19 (79%)	ns*	10 (77%)	18 (75%)	ns*
Ritchie Index, mean ± SD	10.5 ± 8.45	9.71 ± 8.32	ns^	4.25 ± 3.52	1.62 ± 2.28	0.004^
VAS pain (mm), mean ± SD	64.2 ± 19.9	60.8 ± 27	ns^	61.4 ± 31.2	20.1 ± 25.1	0.001^
VAS disease patient (mm), mean ± SD	58 ± 23.6	57.8 ± 24.4	ns^	48.7 ± 27.3	21.6 ± 22.3	0.004^
EGA (mm), mean ± SD	52.7 ± 26.2	54.8 ± 18.3	ns^	36 ± 20.5	17.6 ± 18.5	0.01^
Global Health, mean ± SD	48.6 ± 22.3	63.9 ± 17.3	ns^	35 ± 16.2	17.3 ± 11.8	0.03^
Number of tender joints (0–44), median (range)	11.5 (2–35)	11.5 (0–33)	ns^	6 (1–19)	1 (0–13)	0.002^
Number of swollen joints (0–44), median (range)	5 (1–22)	5.5 (0–13)	ns^	1.5 (0–9)	0 (0–8)	0.02^
Number of tender joints (0–28), median (range)	7 (2–24)	7.5 (0–23)	ns^	3.5 (1–11)	0 (0–4)	0.001^
Number of swollen joints (0–28), median (range)	3.5 (1–20)	5 (0–13)	ns^	1.5 (0–8)	0 (0–8)	0.01^
DAS 28 score, mean ± SD	5.1 ± 0.7	5.2 ± 1.2	ns^	3.6 ± 1.03	2.3 ± 0.9	0.001^
Remission (DAS28 < 2.6), number (%)	-	-	-	2 (16)	16 (68)	0.001
Low disease activity (DAS28 2.6-3.2), number (%)	0 (0)	1 (4.2)		2 (15)	4 (16)	
Moderate disease activity (DAS 28 3.2-5.1), number (%)	10 (77)	11 (45.8)		8 (62)	3 (12)	
High disease activity (DAS28 > 5.6), number (%)	4 (23)	12 (50)		1 (8)	1 (4)	
Good responders, number (%)	-	-	-	0 (0)	1 (4)	
Moderate responders, number (%)	-	-	-	10 (75)	23 (96)	
No responders, number (%)	-	-	-	3 (25)	0 (0)	
Ultrasound Total Synovitis score, mean ± SD	26.5 ± 31.3	25.4 ± 17.4	ns^	14.3 ± 15.6	8.1 ± 6.2	ns ^
Ultrasound Total power Doppler score, mean ± SD	4.6 ± 8.9	2.7 ± 4.2	ns^	0.83 ± 1.32	0.57 ± 1.26	ns^
Ultrasound Total Erosion score, mean ± SD	1.2 ± 2.3	1.4 ± 3	ns^	11.8 ± 6.4	9.6 ± 11.2	ns^

Results are shown as mean ± SD or number (percentage) of patients, as appropriated. ^Mann–Whitney test applied, *chi-squared test applied. P-value <0.05 are considered significant. ESR, erythrocyte sedimentation rate; CRP, C-reactive protein; RF, Rheumatoid Factor; ACPA, anti-citrullinated protein antibodies; VAS, visual analogue scale; EGA, examinator’s global disease activity assessment; DAS28, 28-joint disease activity score.

### Response to RA treatment and clinical remission at 12 months from diagnosis

After 12 months follow-up all participants were in treatment with low doses of corticosteroids (Prednisone < 10 mg/day) and methotrexate (7.5-15 mg/week). Among our study population 91.7% patients were responders to RA treatment (2.7% good and 89% moderate responders) according to the EULAR response criteria. Disease remission (DAS28 < 2.6) was achieved by 48% of patients. The percentage of responders was significantly lower in the subgroup of patients with hypovitaminosis D compared to subjects with normal 25(OH) vitamin D levels at baseline (25(OH) vitamin D < 20 ng/ml: 75% responders (0% good, 75% moderate response) vs 25(OH) vitamin D > 20 ng/ml: 100% responders (4% good, 96% moderate response, p < 0.001).

Similarly, the percentage of patients in remission was significantly reduced in hypovitaminosis D subgroup compared with the normal 25(OH) vitamin D one as showed by clinimetric index DAS28 (16% vs 68%, p < 0.001). The same results were obtained also using other clinimetric indexes such as DAS44, CDAI and SDAI (not shown) thus reinforcing the evidence for the association between hypovitaminosis D and disease activity/response to treatment.

The bivariate correlation analyses showed that the response to RA treatment and the clinical remission after 12-month from RA diagnosis were associated with baseline vitamin D status (Spearman’s coefficient 0.45, p = 0.006; Spearman’s coefficient 0.55, p < 0.001, respectively) and did not correlate with age, gender, BMI, CRP, ESV, DAS28, ACPA and RF positivity at the time of RA diagnosis. Moreover, the multivariate logistic analysis demonstrated that baseline vitamin D status independently predicted the response to RA treatment after adjusting for age, gender and all possible confounders (p < 0.006, see also Additional file 1: Table S1). Notably, in our study population, baseline hypovitaminosis D was associated with an over 3-fold increase (OR: 3.5; 95% C.I.: 1.3-9.5, χ^2^ test applied) in the risk of no response to RA treatment after 12 months follow-up.

The existence of a worse response to treatment in patients with hypovitaminosis D compared to those with normal 25(OH) vitamin D levels at RA onset was also demonstrated by US. Although individual US scores underwent changes that were not significantly different between the two groups, when calculating the difference between US total synovitis scores at baseline and 12-month follow-up, a significant difference was observed between score variation in patients with hypovitaminosis D and the one in patients with normal 25(OH) vitamin D (Variation: −7.1 ± 15.3 vs −20.4 ± 12.9, respectively; p = 0.01).

## Discussion

In this study we demonstrated that the occurrence of hypovitaminosis D in early RA is predictive of a reduced response to treatment and higher disease activity as evaluated after 12 months follow-up. Low vitamin D levels were found in 35% of study population and vitamin D levels were not associated with basal RA activity. So far, conflicting evidence exists regarding the association between vitamin D deficiency and RA susceptibility and prognosis. Although an overall high prevalence of vitamin D deficiency in RA has been largely described, some studies found comparable serum 25(OH) vitamin D concentrations between patients with RA and healthy controls [16,17]. Gheita TA et al. recently found reduced 25(OH) vitamin D levels in RA patients compared with controls and that, among RA patients, vitamin D was significantly lower in subjects with fibromyalgia syndrome in addition to RA [15]. Other studies evaluated prospectively the risk of RA in relation to vitamin D intake, but again the results are conflicting [18,30,31].

So far, evidence of a relationship between vitamin D serum levels and disease activity in early RA patients is limited to a report on a negative association [21,32] between serum vitamin D at baseline and HAQ score after 12 months follow-up, but neither RA activity nor response to treatment were investigated. Cross-sectional studies on the association between baseline 25(OH) vitamin D levels and disease’s activity in early RA patients did not obtain univocal results [19-21].

In our study, as expected, we observed an overall significant reduction of RA activity, evaluated by clinimetric, laboratory and US parameters, after 12 months of treatment. In fact, the outcome of the disease has improved considerably in recent years with the availability of effective therapies and the recognition that early intensive treatment strategies result in better outcomes. Interestingly, at the end of follow-up we found that patients with hypovitaminosis D exhibited significantly higher disease activity and reduced percentage of remission and lower response to treatment compared with those with sufficient 25(OH) vitamin D levels at baseline, suggesting a role of vitamin D in disease progression and prognosis. Over the past decade the immunomodulatory role of vitamin D has become more defined. Experimental data show that vitamin D metabolites play a modulatory role on T cell proliferation and dendritic cell function via VDRs expression on these cells [33]. Notably, the expression of VDRs was also demonstrated on activated lymphocytes, synoviocytes, macrophages, and chondrocytes in the course of RA [33]. In murine models of collagen-induced arthritis, vitamin D supplementation prevents both the onset and progression of arthritis [8]. Further support for a role of VDR/vitamin D system in the pathogenesis of RA is also provided by epidemiological studies showing the existence of an association between VDR Fok1polymorphism and RA susceptibility [34,35].

One limitation of the present study is the small number of patients; another limit is the short duration follow-up, as 12 months may not be enough to ascertain whether vitamin D status at baseline has long term effects on RA activity, particularly referring to structural damage. Moreover, as no previous data are available on clinical remission/response to treatment after a 12 month follow-up in relation to vitamin D status at RA onset, we just performed a post-hoc sample size calculation, confirming the statistical power of our findings.

Our study however, has several strengths: it is the first longitudinal study investigating the role of vitamin D status in predicting RA activity and response to treatment in early and DMARDs-naïve RA and evaluates for the first time the prognostic value of vitamin D status on US parameters in RA, such as synovitis, p-D and bone erosion US scores.

## Conclusions

In conclusion, our study demonstrates that hypovitaminosis D at RA onset is predictive of a reduced response to treatment in early RA after 12 months of follow-up. These results provide further support to the immunomodulatory action of vitamin D in inflammatory arthritis and suggest a potential prognostic role of vitamin D status at diagnosis in RA evolution. Serum vitamin D measurement and possibly vitamin D supplementation should be considered an additional option in the management of early RA patients.

## Additional file

Additional file 1: Table S1. Multivariate logistic analysis.

## Abbreviations

1,25(OH)2D: 1,25-dihydroxyvitamin D
ACPA: Anti-citrullinated protein antibodies
CRP: C-reactive protein
DAS28: 28-joint disease activity score
DMARDs: Disease-modifying anti-rheumatic drugs
EGA: Examinator’s global disease activity assessment
ESR: Erythrocyte sedimentation rate
PFR: Peak frequency ratio
RA: Rheumatoid arthritis
RF: Rheumatoid Factor
SE: Synovial effusion
SH: Synovial hypertrophy
US: Ultrasound
VAS: Visual analogue scale
VDR: Vitamin D receptor
