# Supplementary material for: Hypovitaminosis D in recent onset rheumatoid arthritis is predictive of reduced response to treatment and increased disease activity: a 12 month follow-up study
Source: BMC Musculoskelet Disord. 2015 Mar 15;16:53. doi: 10.1186/s12891-015-0505-6 (PMC4373034; doi:10.1186/s12891-015-0505-6)
Supplement: Additional file 1: Table S1. — Multivariate logistic analysis. [file 12891_2015_505_MOESM1_ESM.docx]

| **Equation Variables** | | | | | | | | | |
| --- | --- | --- | --- | --- | --- | --- | --- | --- | --- |
|  | | B | E.S. | Wald | df | Sig. | Exp(B) | 95% IC for EXP(B) | |
|  |  |  |  |  |  |  |  | Inferior | Superior |
|  | **VitD** | **3,959** | **1,450** | **7,451** | **1** | **,006** | **52,409** | **3,054** | **899,478** |
|  | BMI | ,021 | ,148 | ,020 | 1 | ,888 | 1,021 | ,764 | 1,364 |
|  | Gender | 3,996 | 4,748 | ,709 | 1 | ,400 | 54,396 | ,005 | 598124,635 |
|  | Age | -,031 | ,056 | ,303 | 1 | ,582 | ,969 | ,868 | 1,083 |
|  | CRP | ,069 | ,096 | ,516 | 1 | ,473 | 1,072 | ,887 | 1,295 |
|  | RF + | -,955 | 2,214 | ,186 | 1 | ,666 | ,385 | ,005 | 29,520 |
|  | ACPA + | -2,858 | 3,221 | ,788 | 1 | ,375 | ,057 | ,000 | 31,621 |
|  | DAS28 | 2,058 | 1,531 | 1,808 | 1 | ,179 | 7,832 | ,390 | 157,370 |
|  | ESR | -,186 | ,100 | 3,430 | 1 | ,064 | ,830 | ,682 | 1,011 |
|  | Constant | -6,158 | 6,228 | ,978 | 1 | ,323 | ,002 |  |  |
|  | | | | | | | | | |

**Supplementary data**

Multivariate logistic analysis. Response to RA treatment is considered as dependent variable.
